# Supplementary material for: Use of the Non-Pneumatic Anti-Shock Garment (NASG) for Life-Threatening Obstetric Hemorrhage: A Cost-Effectiveness Analysis in Egypt and Nigeria
Source: PLoS One. 2013 Apr 30;8(4):e62282. doi: 10.1371/journal.pone.0062282 (PMC3640005; doi:10.1371/journal.pone.0062282)
Supplement: Technical Appendix S1 — Description of methods for collecting input data, calculating cost-effectiveness, and performing sensitivity analyses. (DOC) [file pone.0062282.s001.doc]

**Technical Appendix S1. Cost-effectiveness calculations**

***Analysis overview***: Calculations were performed in Excel 2004. The analysis was

stratified by country, study phase and mean arterial pressure (MAP) on study admission.

MAP was calculated by: ((2*diastolic blood pressure) + systolic blood pressure)/3). For each country, we used observed proportions of initial shock level (mild: mean arterial pressure [MAP] > 60mmHg; severe: MAP ≤ 60mmHg) to define a standard population of 1,000 women presenting in shock. We examined three intervention scenarios: no women in shock receive the NASG, only women in severe shock receive the NASG, and all women in shock receive the NASG.

***Costs***: The costs of uterotonics, misoprostol (200ug), ergometrine (0.2mg) and oxytocin

(10iu), and one liter of bleach for NASG disinfecting were estimated by using the

pharmacy nearest to the hospital as a reference. In Nigeria, the cost of oxytocin obtained

from one hospital, UCH, was almost 40x greater than the other facility and all estimates

in the literature; in absence of a reliable confirmation of this cost, we used a standardized

value from the literature.[1] In Egypt, we used a weighted average of the costs for

ergometrine and oxytocin to estimate total costs for uterotonics. For blood transfusion,

providers were asked to report the unit price of a 500ml blood transfusion, in addition to

any transaction costs and laboratory fees such as, cross-matching and cost of testing for

communicable diseases (human immunodeficiency virus (HIV), Hepatitis B & C).

Emergency hysterectomy costs included a facility fee, provider fee, materials (disposable

and non-disposable) and medications, if applicable.

Training costs for obstetric care providers (nurses, nurse-midwives and physicians) at

study sites were calculated by the project manager at the University of California, San

Francisco (UCSF) with receipts from the original training sessions and included cost of

local trainer salary, national travel for the trainer, local transport, refreshments and room

and equipment rental. The model also considered the number of staff that had to be

trained, annual deliveries, and the number of training sessions per day with a maximum

number of twenty individuals trained per session. Clinically, providers were trained to

apply the garment, monitor vital signs, level of consciousness, urine output, blood

loss, apply standard protocols for vaginal procedures and/or hemostatic surgeries,

and correct NASG removal. Providers, public health personnel, were paid their normal

salary during the hour-long training, but may have missed income from elective

procedures or private practice income. The collective physician time spent was assumed

to be 20 hours at a wage of $15.44 per hour. [2] Although each provider only needs to be

trained once over the course of a lifetime, we assumed in this analysis that the training

was valid for ten years.

The manufacturer price and the cost associated with cleaning the NASG with bleach

between uses was collected. Cost of the NASG was determined by dividing the

manufacturer’s suggested retail price by a standard number of forty uses determined by

prior pilot studies and addition of cleaning costs.[3] The bleach dilution factor for garment disinfection is a 0.05% bleach solution, with 50ml bleach in 4,950ml water and a total of2L bleach required over the NASG lifetime. To account for inaccurate dilution, theft or missing bleach bottles, the amount of bleach was doubled in calculations. Investigators were asked to provide daily wages for cleaning staff, which ranged from $0.13-$1.52 per eight hour shift. With only one or two garments used per day at the highest volume facilities, the labor cost of cleaning the garment were estimated to be less than $0.01/ day and was not included in this analysis.

Collected costs were compared to WHO CHOICE regional estimates. For example, the

CHOICE estimate for hysterectomy, one of the most significant analysis costs, was

expected to be lower than study estimates because it was calculated in 2000 I$. The

CHOICE regional estimates were similar to costs provided at Assuit and Katsina

Hospital, but were significantly lower than costs at the university hospitals, El Galaa and

UCH. We were unable to collect costs for two sites in northern Nigeria because the study

had closed these sites prior to this analysis. As a result, we made estimates using costs

from proximate hospitals that had similar provider and resource access profiles. One

hospital, MMSH, was a government hospital and had similar resource availability to

Katsina General Hospital, while the other, AKTH, was a teaching hospital, with a cost

and resource profile more similar to UCH.

***Cost-effectiveness calculations***: To calculate baseline values, disability-adjusted life years (DALYs) were calculated as the sum of the total years of life lost due to mortality (YLL) and years of life lost due to disability (YLD) (Table 4; YLD/YLL not shown). YLL was calculated by discounting the difference of the average age of maternal death in our study and the age-adjusted female life expectancy.[4] YLD included anemia, infertility secondary to hysterectomy and severe morbidities (heart failure, renal failure, cerebral impairment and motor deficits).[5] YLD for severe anemia, heart failure, acute renal failure and acute respiratory distress was calculated with a standard disability weights from the literature assuming durations less than a year because no discharge follow-up information was available to assess the sequalae, if any, of these peripartum injuries.[6] Infertility’s disability weight was discounted at 3% over average national reproductive spans available in the literature from the hysterectomy cases indicated for a primary uterine atony diagnosis. The weights for cerebral impairment and motor deficits were discounted for 3% over the average woman’s lifetime due to the poor or non-existent rehabilitation options.

The incremental cost-effectiveness ratio (ICER), the change in costs divided by the

change in DALYs, was calculated to compare the NASG intervention with standard care. In the text, "Dominant" means cheaper and better health outcomes than previous intervention scenario, while "Dominated" means more costly and worse outcomes than previous scenario. In the two incremental Egyptian scenarios, there were net-savings and DALYs averted which resulted in negative ICERs,, which were not reported because they have no intrinsic meaning.[7] These intervention scenarios were labeled “Dominant” scenarios. In Nigeria, providing the NASG incrementally for all women as opposed to women in severe shock only resulted in a “Dominated” scenario. However, when the NASG was provided for all women and compared to the “No NASG” scenario, there was an ICER of $7.97. As explained in the text, ICERs are presented in Table 4 for the severe shock group in Nigeria.

***Sensitivity analysis***: All sensitivity analyses were performed from the perspective of the

uterine atony-only hysterectomy group comparing the health costs and outcomes of “No NASG” standard care for all women in shock vs. the NASG for women in severe shock only. For the one-way sensitivity analysis, we used an interval for the variables that was wide enough for the assumptions of the model to be credible. Cost outcomes were assessed assuming 50% variance, with the exception of the NASG low value, $53.76.[3] Health outcomes, such as morbidity, mortality, emergency hysterectomy and severe anemia were also assessed using 20% variance for the rare outcomes of mortality and severe morbidity and assuming 50% variance for severe anemia and infertility. The variance, or health effect, indicates the risk of the event (i.e. mortality) compared to the base case. Therefore, an effect of 0.80 on mortality indicates a 20% decrease in mortality risk in the “NASG for severe shock only” scenario. After a high or low value had been calculated for each variable, the value (or the scale factor multiplied by the base value) was substituted in each original cost or DALY cell, and the change in package cost or change in DALY from intervention to standard care analysis was documented and corresponding ICERs were calculated. The outputs of the sensitivity analysis for cost and DALY values were then compared to baseline values.

***Differences in study sites***: While patients in both countries were low income and using

the public health care system, the health system contexts, health care infrastructures, and

human and capital resources differed. For example, Egypt’s life expectancy for a female

was 70 years in 2010 while Nigeria’s was 52.[4] Further, Egypt’s MMR is 43/100,000

compared to Nigeria’s MMR of 608/100,000 births.[8] In fact, while Egypt is one of the

only countries that met MDG5, Nigeria is one of six countries that contributed to 50% of

all maternal deaths.[8]

**References**

1. Seligman BL. Economic assessment of interventions for reducing postpartum

hemorrhage in developing countries. Bethesda: Abt; 2006.

2. World Health Organization. Making Choices in Health: WHO Guide to Cost Effectiveness Analysis. Geneva: 2003.

3. World Health Organization (WHO). Compendium of new and emerging health technologies Available: [http://whqlibdoc.who.int/hq/2011 /WHO_HSS_EHT_DIM_11.02_eng.pdf](http://whqlibdoc.who.int/hq/2011/WHO_HSS_EHT_DIM_11.02_eng.pdf). Acessed 20 December 2011.

4. World Health Organization (WHO). Global Health Observatory Data Repository: Life tables. Available: <http://apps.who.int/ghodata/?vid=720>. Accessed 11 December 2011.

5. Miller S, Fathalla MM, Ojengbede OA, Camlin C, Mourad-Youssif M,

Morhason-Bello IO, et al. (2010) Obstetric hemorrhage and shock management: using

the low technology Non-pneumatic Anti-Shock Garment in Nigerian and

Egyptian tertiary care facilities. BMC Pregnancy Childbirth. 10:64.

6. Murray C. (1996) The Global Burden of Disease. Cambridge: Harvard Press.

7. Gold M SJ, Russell LB, Weinstein MC (editors). (1996) Cost-effectiveness in health and medicine. New York: Oxford University Press, 425p.

8. Hogan MC, Foreman KJ, Naghavi M, Ahn SY, Wang M, Makela SM, et al. (2010)

Maternal mortality for 181 countries, 1980-2008: a systematic analysis of

progress towards Millennium Development Goal 5. Lancet. 375(9726):

1609-23.
